# Rapid ultrasensitive detection platform for antimicrobial susceptibility testing

**DOI:** 10.1371/journal.pbio.3000291

**Published:** 2019-05-30

**Authors:** Mehmet F. Cansizoglu, Yusuf Talha Tamer, Michael Farid, Andrew Y. Koh, Erdal Toprak

**Affiliations:** 1 Green Center for Systems Biology, University of Texas Southwestern Medical Center, Dallas, Texas, United States of America; 2 Department of Biomedical Engineering, Johns Hopkins University, Baltimore, Maryland, United States of America; 3 Department of Pediatrics, University of Texas Southwestern Medical Center, Dallas, Texas, United States of America; 4 Department of Microbiology, University of Texas Southwestern Medical Center, Dallas, Texas, United States of America; 5 Department of Pharmacology, University of Texas Southwestern Medical Center, Dallas, Texas, United States of America; The Pennsylvania State University, UNITED STATES

## Abstract

Rapid detection and phenotyping of pathogenic microbes is critical for administration of effective antibiotic therapies and for impeding the spread of antibiotic resistance. Here, we present a novel platform, rapid ultrasensitive detector (RUSD), that utilizes the high reflectance coefficient at high incidence angles when light travels from low- to high-refractive-index media. RUSD leverages a principle that does not require complex manufacturing, labeling, or processing steps. Utilizing RUSD, we can detect extremely low cell densities (optical density [OD] ≥ 5 × 10^−7^) that correspond to approximately 20 bacterial cells or a single fungal cell in the detection volume, which is nearly 4 orders of magnitude more sensitive than standard OD methods. RUSD can measure minimum inhibitory concentrations (MICs) of commonly used antibiotics against gram-negative and gram-positive bacteria, including *Staphylococcus aureus*, *Pseudomonas aeruginosa*, and *Escherichia coli*, within 2 to 4 h. Here, we demonstrate that antibiotic susceptibility tests for several pathogens can rapidly be performed with RUSD using both small inoculum sizes (500 cells/mL) and larger inoculum sizes (5 × 10^5^ cells/mL) used in standard antibiotic susceptibility tests. We anticipate that the RUSD system will be particularly useful for the cases in which antibiotic susceptibility tests have to be done with a limited number of bacterial cells that are available. Its compatibility with standard antibiotic susceptibility tests, simplicity, and low cost can make RUSD a viable and rapidly deployed diagnostic tool.

## Abstract 2

This article presents a novel diagnostic platform, the rapid ultrasensitive detector (RUSD), able to detect as few as 20 bacterial cells or a single fungal cell, and allowing the rapid measurement of antibiotic susceptibility.

## Introduction

Optimal treatment of infectious diseases requires early detection of pathogenic microbes [1]. Despite advances in medical technology, both detection and characterization of pathogenic microbes can still take up to several days [2–4]. In many cases, clinicians are relegated to starting empiric antibiotic therapies that are typically broad in coverage and may have deleterious sequelae such as inducing gut microbiome dysbiosis and potentially exacerbating the ongoing antibiotic resistance crisis [5]. Therefore, there is an urgent need for a rapid, affordable, and highly sensitive detection platform that can significantly reduce the time needed for antibiotic susceptibility determination and enable optimized, targeted therapy selection sooner [1,2,6].

**Fig 1:**
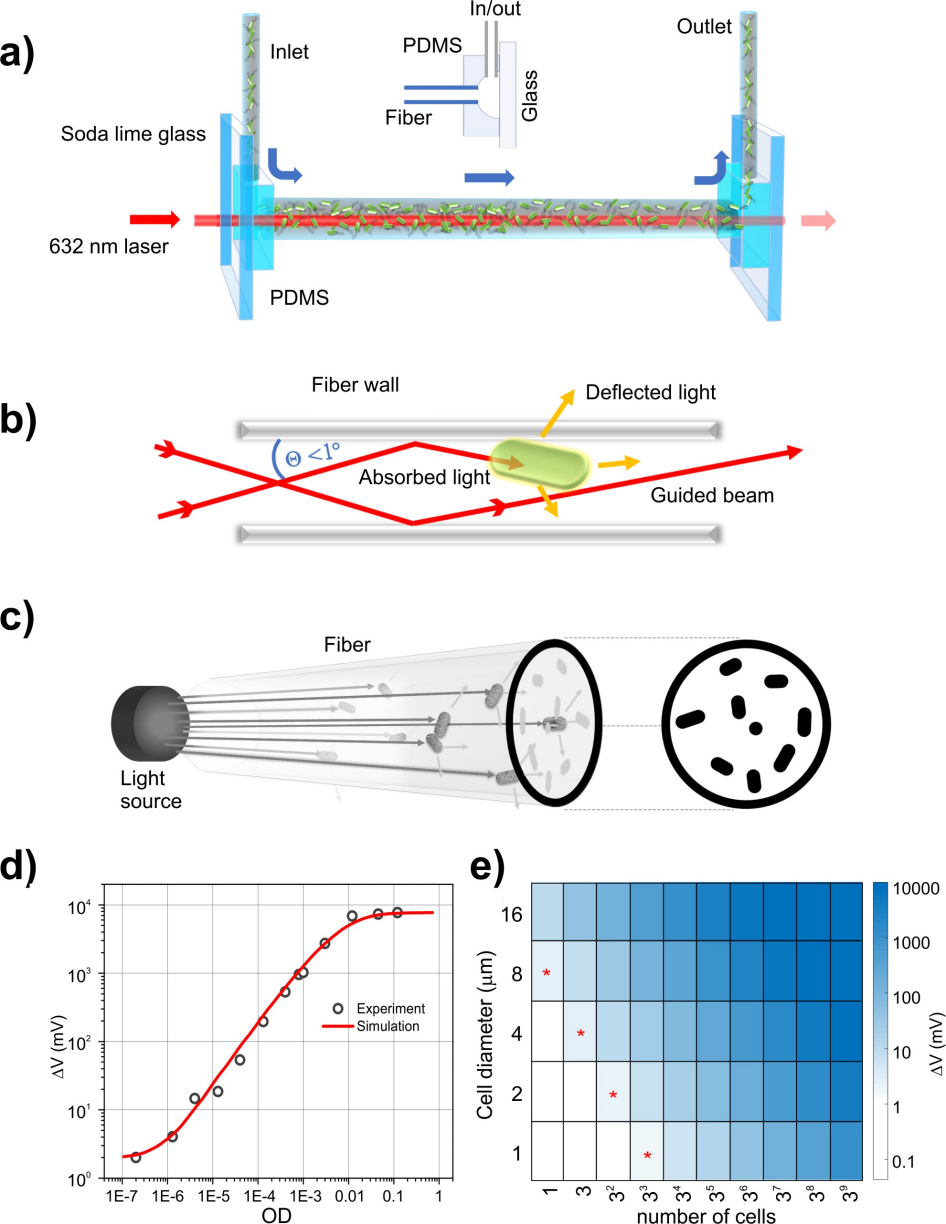
RUSD. (a) Optical schematics of the RUSD apparatus, which utilizes the high reflectance coefficient of light at the interface between thin and dense media (water and glass) at high incidence angles. (b) The focal distance of the laser is set such that the incidence angle (θ) of the laser at the fiber wall remains > 89°. At θ > 89°, the reflectance coefficient of laser light is R > 0.82 for both s and p polarizations of light, allowing the laser beam to travel in liquid and reflect multiple times within the fiber. (c) During travel, any photon encountering a cell or a particle is either absorbed or scattered, losing the angular condition to be guided by the fiber (i.e., θ > 89^o^), and any cell on the light path reduces the total intensity proportional to its cross-sectional area and creates a 2D shadow-projection image on the optical detector. The current from the reverse-biased optical detector is passed through a resistor in which the potential difference is converted to a signal for data acquisition. (d) The voltage signal as a function of cell density. Simulation data based on the mathematical model described here and actual RUSD exhibit a high degree of concordance (R^2^ = 0.97) up to OD > 10^−3^ and (R^2^ = 0.93) for the whole data range used here. (e) Signal strength versus number of cells and cell dimensions in RUSD: As the cell size increases, signal strength is multiplied proportionally to the cell cross-sectional area. The cell shape is taken as a square pattern with the specified dimensions. The region marked with red asterisks indicates the boundary of detection at which the signal is above the 1 mV limit. RUSD signal rapidly saturates over approximately OD 0.1 as cells optically block the fiber cross section. OD, optical density; PDMS, polydimethylsiloxane; RUSD, rapid ultrasensitive detector.

Conventional culture-based assays require microbial growth at high cell densities and are thus inherently slow [3,4]. Here, we introduce rapid ultrasensitive detector (RUSD), a label-free and low-cost fiber-based platform (see Materials and methods). The hollow fiber in the RUSD apparatus is utilized as an optofluidic channel, which serves as a selective waveguide for laser light and as a detection and/or growth chamber for microbial cells. Microbial cells flow through the channel either by continuous circulation that includes a computer-operated pump and a degassing/bubble trap mechanism to avoid bubble formations, or simply by manual injection via a syringe (Fig 1A). RUSD takes advantage of the loss of reflectivity within the hollow fiber because of both optical absorption and scattering events. Photons that hit obstacles on the travel path are deflected and violate the condition for high reflectivity. The laser beam is coupled into the hollow core of the fiber, together with cells in growth media (index of refraction [n_i_] = 1.33). The laser beam is focused to the entrance of the fiber such that the incidence angle of the laser at the fiber wall remains at a grazing of ≥89°, which keeps the reflectance coefficient R > 0.82 for both s and p polarizations of light (Fig 1B). Photons that hit obstacles on the travel path, however, lose the condition for high reflectivity through mainly scattering or absorption events and are selectively filtered out along the hollow fiber waveguide. This creates a 2D cross-sectional projection shadow of the cells dispersed inside a 3D volume at the end of fiber (Fig 1C). Total light lost during travel is estimated by $A=I_{0}(\sum_{i=1}^{n}a_{i}/a_{fiber})\approx I_{0}n\pi r^{2}/a_{fiber}$ (Equation 1), where *I*_0_ is the intensity of light entering the fiber, a_i_ is the cross-sectional area of the i^th^ cell, a_fiber_ is the cross-sectional area of the fiber (i.e., approximately 0.2 mm^2^), n is the cell number, and r is the average radius of a cell (assuming spherical cell shape, i.e., approximately 1 μm for bacteria). This equation remains valid when the total cross-sectional area of cells is less than the cross-sectional area of the fiber. The light intensity at the end of the fiber is measured through a photodiode, in which the difference in reference to blank media measurement (ΔV) is converted to optical density (OD) readings (i.e., OD = 1 corresponds to approximately 5 × 10^8^ cells per milliliter of *E*. *coli* culture) using a calibration curve (Fig 1D).

The physical model explaining the working principle of RUSD suggests that the detector response (ΔV) is proportional to the ratio between projected cell area and fiber cross-sectional area (Equation 1). Thus, increased cell size or decreased fiber diameter enhances sensitivity, making it possible to detect larger cells (e.g., eukaryotic cells) at lower densities. At a sensitivity threshold of ΔV = 1 mV, which is typical for low-cost electronics, the detection system is estimated to be capable of sensing about 27 spherical cells of 1 μm radius, or a single spherical cell of 8 μm diameter in a 500 μm diameter, 40 cm long (L) fiber setup (Fig 1E). The projection coverage of the cells also increases linearly with the encapsulated fiber volume, laying out a simple (ΔV α L) relation with the length of the optical fiber. Signal strength (ΔV) in RUSD behaves in accordance with ΔV α 1/R^2^, where R is the inner diameter of the hollow core. Thus, a decrease in fiber diameter will enhance the sensitivity per cell with the cost of reducing the detection volume. The signal in the detection system model saturates at relatively low cell densities (approximately OD 0.01 for *E*. *coli* cells, Fig 1D) because the narrow cross section of the fiber is optically blocked by randomly distributed cells in 3D space.

On the other hand, in the conventional absorption-based OD measurements, which employ the Beer-Lambert relation, absorption is given as *A* = *I_o_*(1−*e^−μl^*) (Equation 2), where μ is the attenuation coefficient, and *l* is the length (thickness) of the specimen. For a well-dispersed absorbing species, μ is also dependent on the volumetric concentration. In a nonuniform absorption case, as in liquid bacterial cultures, light absorption per single cell is correlated with the size of the cell and hence is almost undetectable. Therefore, in order to increase the sensitivity of absorption-based methods to that of RUSD, the optical path length would have to be increased approximately 10^3^–10^4^ times (e.g., 100-meter-long sample thickness), which is not feasible.

## Results

To determine the sensitivity of the RUSD platform, we first calibrated our system using bacterial (*E*. *coli*, *S*. *aureus*, and *P*. *aeruginosa*) and fungal (*Saccharomyces cerevisiae*, *Candida glabrata*) cells. Overnight cultures were serially diluted, and the voltage signals generated (ΔV) in RUSD were recorded. The cell densities of diluted cultures were also confirmed by colony forming unit (CFU) counts conducted for the very same bacterial cultures (1 OD ≈ 5 × 10^8^ CFU/mL for all tested bacteria) and fungal cultures (1 OD ≈ 2.2 × 10^7^ CFU/mL for *S*. *cerevisiae* and 1 OD ≈ 1.25 × 10^7^ CFU/mL for *C*. *glabrata*). The measured ΔV values and corresponding OD values were linearly correlated on a logarithmic scale on both axes (S1A Fig). Based on this linear correlation, we used a power function (log_10_[Δ*V*] = *a* + *b* log_10_[*OD*]) to fit our data and converted voltage readings to OD values and cell counts. In our experimental settings, for bacteria and fungi, the value of *a* was typically between 2.9 and 3.1, whereas *b*, the slope of the calibration curve, was found to be between 0.9 and 1.1. The limit of detection (LOD) [7,8] attained at the standard RUSD setup (400 mm long fiber with 500 μm inner diameter) was OD ~5 × 10^−7^ for bacteria (S1B Fig), which corresponds to approximately 250 bacterial CFU/mL. The limit of quantification (LOQ, [7,8] minimum level of signal after which detector response increases proportional with the cell density) for RUSD was OD ≥ 1.5 × 10^−6^ for bacteria (S1B Fig), which corresponds to approximately 750 bacterial CFU/mL. Given that our detection volume is roughly 80 μL, we can detect as low as about 20 bacterial cells, which we measured to be about 10^4^-fold more sensitive than our commercial plate reader (Tecan M200 Pro). We showed that RUSD can detect a single fungal cell (spherical *S*. *cerevisiae* or *C*. *glabrata* with 5–6 μm diameter). All measurements with bacterial cells were done in lysogeny broth (LB) media. The system is, as all optical methods are, limited by the transparency of the medium to the laser light used. Using a more transparent media such as minimal M9 media improves the sensitivity of the instrument by increasing the signal-to-noise ratio, especially in longer fiber setup schemes, decreasing LOD down to about 2 × 10^−7^, which corresponds to approximately 8–10 bacterial cells. For practical MIC measurement purposes, any transparent media commonly available can be utilized in measurements.

Depending on the growth conditions and cell type, conventional approaches to detect microbial growth may require several hours to days. In laboratory or clinical settings, the time required for quantitatively measuring bacterial growth largely depends on the inoculum size of bacteria and growth conditions. For *P*. *aeruginosa* (PAO1), *S*. *aureus* (RN4220), *E*. *coli* (MG1655), and an *E*. *coli* clinical isolate (ET-CI28), we first quantified the lag time before statistically significant growth as a function of inoculum size using a microplate reader (S2 Fig). For each microbial strain, the lag time was defined as the first time point in which cell density exceeded the LOQ for our plate reader (LOQ = 0.02, OD_600_). When the initial OD of the bacterial culture is 10^−6^ (nearly equal to the LOQ of RUSD), the lag times for the tested microbial strains ranged between 6 and 10 h. Long lag times hinder clinical applications such as antibiotic susceptibility testing (AST) and thus necessitate using large microbial inoculums (i.e., 5 × 10^5^ CFU/mL and OD_600_ > 0.001).

**Fig 2:**
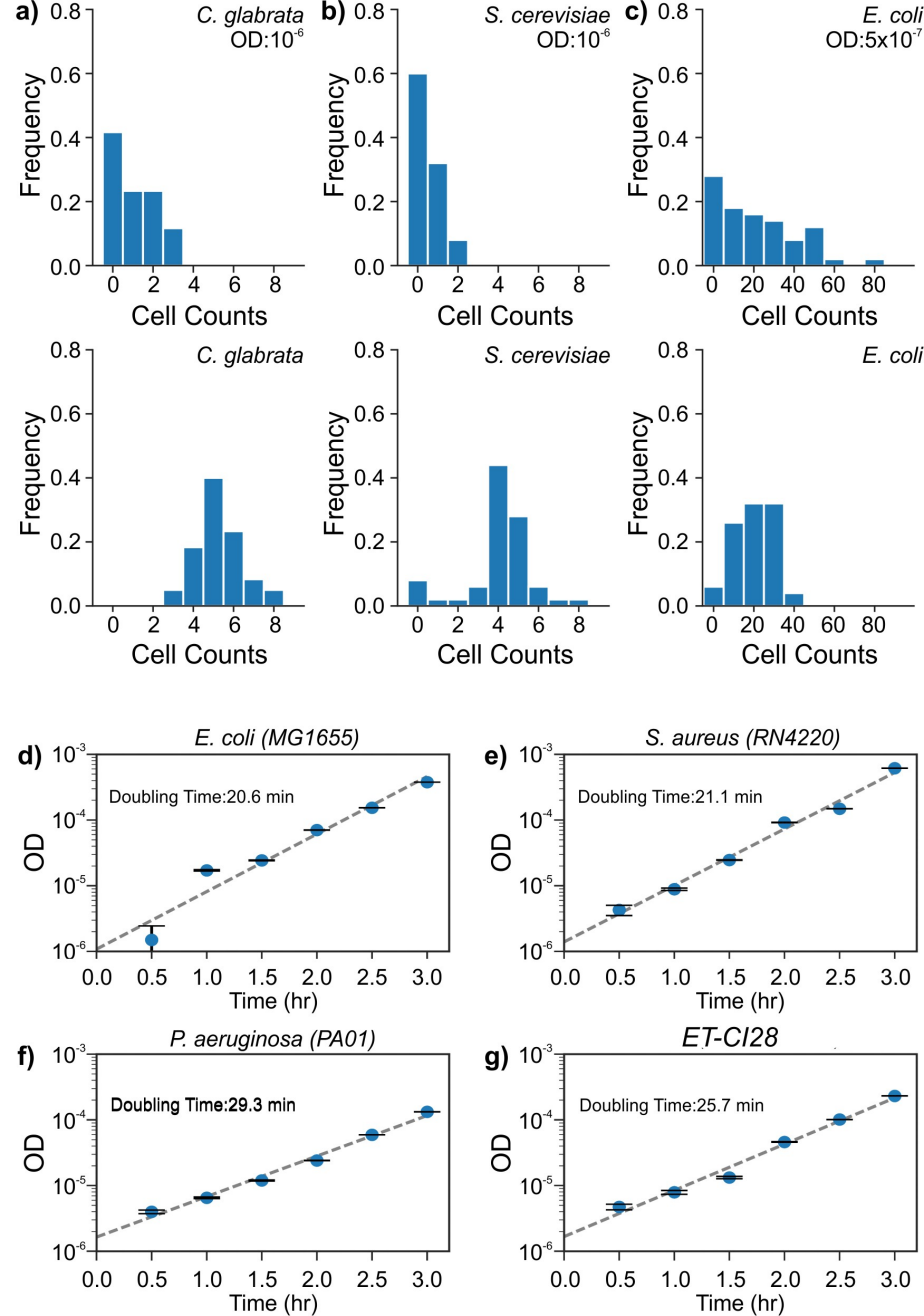
RUSD detects extremely low bacterial and fungal cell counts. (a) Top panel: Counts for *C*. *glabrata* at approximately OD 10^−6^ displays a Poisson distribution with counts of 0, 1, 2, and 3 with a mean value of about 1. Bottom panel: Increasing the yeast cell density shifts distribution toward a normal distribution with a mean value of about 5.3 cells. (b) *S*. *cerevisiae* at an OD nearly 10^−6^ (top panel) results in a Poisson distribution with counts of 0, 1, and 2 and a mean value of approximately 0.48; (lower panel) a slight increase of cell density moves distribution toward a normal distribution with a mean value of approximately 4 cells. (c) Counts for *E*. *coli* culture at (top panel) an OD of approximately 5 × 10^−7^, yields about 25 cells; (lower panel) increasing the bacterial cell density slightly shifts distribution toward a normal distribution with a similar mean value of about 24 cells with smaller standard deviation, which shows the sensitivity limit of the device. ODs of bacterial cultures grown in LB (started from OD roughly 5 × 10^−7^) and recorded by RUSD: (d) *E*. *coli* (MG1655), (e) *S*. *aureus* (RN4220), (f) *P*. *aeruginosa* (PAO1), and (g) an *E*. *coli* clinical isolate (ET-CI28). (The data for Fig 2 can be found in S1 Data). LB, lysogeny broth; OD, optical density; RUSD, rapid ultrasensitive detector.

In contrast, RUSD detects bacterial growth within 1 h. For all 4 bacterial strains, rapid growth was exhibited even at the very initial stages in measurements taken at intervals of about 30 min (Fig 2D, 2E, 2F and 2G). The doubling times were 29.3 min for *P*. *aeruginosa* strain PAO1, 21.1 min for *S*. *aureus* RN4220, 20.6 min for *E*. *coli* MG1655, and 25.7 min for the *E*. *coli* (ET-CI28) isolate. We were also able to use the fiber in the RUSD apparatus as a growth chamber. When we injected *E*. *coli* (MG1655) culture with an initial OD of 10^−5^ inside the fiber (37°C, LB media, no mixing or shaking), we observed bacterial growth within the first 30 min and exponential growth within the first 2.5 h, for which the doubling time was approximately 28 min (S1C Fig). Being able to detect microbial growth at such low cell densities will substantially expedite the time required for AST.

Existing standard antibiotic susceptibility tests are primarily based on 3 methods: (1) disk diffusion assays and (2) liquid-based minimum inhibitory concentration (MIC) measurements in microtiter plates and (3) linear gradient diffusion MIC strips (eTest). The clinical efficiencies of these methods are limited by requirement for overnight incubation of the bacterial cultures [9]. To determine whether the RUSD platform could expedite the antibiotic resistance detection process, we developed a simple assay called “in Fiber Antibiotic Susceptibility Testing” (iFAST). Briefly, we either grow bacterial cells in flasks and periodically monitor growth using RUSD or utilize the hollow fiber as a growth chamber. As a proof of concept, we conducted antibiotic susceptibility assays for levofloxacin, amikacin, and piperacillin-tazobactam (P-T) for strains of *E*. *coli* (MG1655, ET-CI28), *P*. *aeruginosa* (PAO1), and *S*. *aureus* (RN4220), as indicated, using RUSD and microbroth dilution approach (initial cell density = approximately 500 cells/mL or OD of approximately 10^−6^).

**Fig 3:**
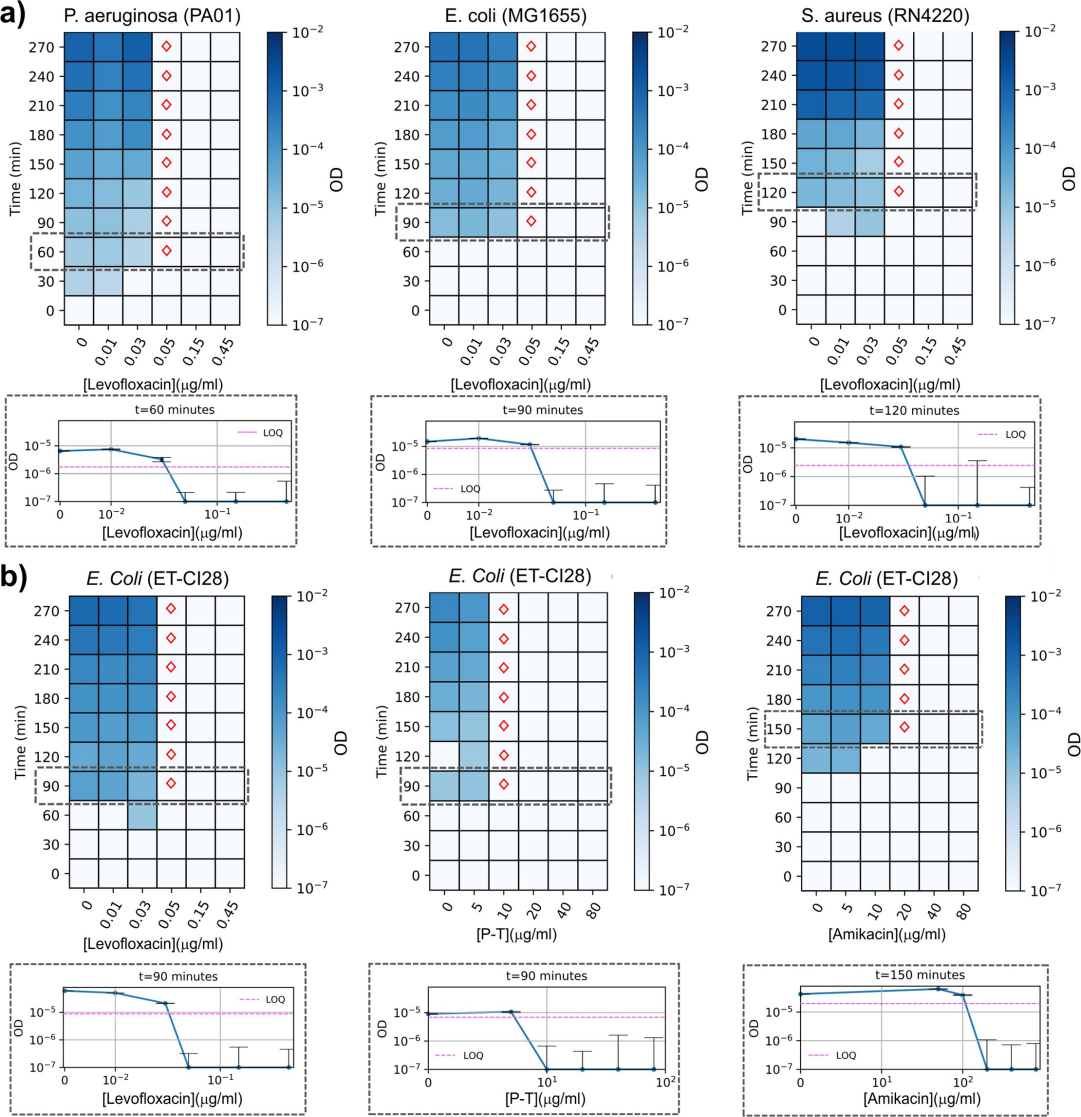
iFAST yields antibiotic resistance profile of pathogenic bacteria in 1 to 3 h. Bacterial cultures with initial OD of approximately 10^−6^ were grown in different concentrations of antibiotics, and their densities were periodically monitored in RUSD. Heat map plots show growth of bacteria as a function of time and antibiotic dosage. Colored bars indicate ODs of bacterial cultures. Red diamonds indicate MIC values measured at different time points at which bacterial growth is statistically significant (OD > LOQ). Below each heat map plot, a dose-response curve is provided for the first statistically significant MIC measurement. A calibration curve is generated for every strain before every measurement, and LOQ values are shown by using dashed magenta lines. (a) Time-resolved dose-response measurements for laboratory strains of *P*. *aeruginosa* (PAO1), *E*. *coli* (MG1655), and *S*. *aureus* (RN4220) in levofloxacin, a DNA gyrase inhibitor. (b) Time-resolved dose-response measurements for a clinical *E*. *coli* isolate (ET-CI28) in levofloxacin, P-T (a beta-lactam cell-wall synthesis inhibitor and beta-lactamase inhibitor), and amikacin (an aminoglycoside). (The data for Fig 3 can be found in S2 Data). iFAST, in Fiber Antibiotics Susceptibility Testing; LOQ, limit of quantification; MIC, minimum inhibitory concentration; OD, optical density; P-T, piperacillin-tazobactam; RUSD, rapid ultrasensitivity detector.

For *E*. *coli* strains (MG1655 and ET-CI28), *P*. *aeruginosa* (PAO1), and *S*. *aureus* (RN4220), RUSD revealed the levofloxacin dose response within about the first 2 h of the iFAST assay, with constant MIC values of 0.05 μg/mL over time (range 60–270 min) for both *E*. *coli* strains and 0.5 μg/ml for both PAO1 and RN4220. (Fig 3). The MIC values for levofloxacin measured by the robotic platform for both *E*. *coli* strains, *P*. *aeruginosa*, and *S*. *aureus* were 0.04 μg/mL, 0.3 μg/mL, and 0.2 μg/mL, respectively. For the clinical *E*. *coli* isolate (ET-CI28), the MIC values for amikacin and P-T obtained by iFAST assays were 20 and 10 μg/mL, respectively. Corresponding MIC values by the robotic platform were 10 μg/mL and 3 μg/mL, which required 7 to 14 h (S1 Movie, S3 Fig). The minor differences between the MIC values measured by RUSD and our robotic platform are likely due to nonidentical experimental conditions used for these assays. In order to confirm that as the cause for those differences, we grew *E*. *coli* cells (MG1655 and ET-CI28) in large flasks using increasing concentrations of antibiotics (P-T and amikacin, respectively) and periodically measured cell densities in both RUSD and a spectrophotometer (S4A Fig and S4B Fig). In both cases, the MIC values measured using both RUSD and spectrophotometer were concordant, suggesting the initial differences in MIC values we observed were unlikely to be due to differences in detection method. Also, the time required to obtain MIC values using RUSD was about 5 h shorter than the time required using the spectrophotometer. In summary, for several bacterial strains and antibiotics, RUSD reduced the time for susceptibility characterization by 5 to 12 h.

**Fig 4:**
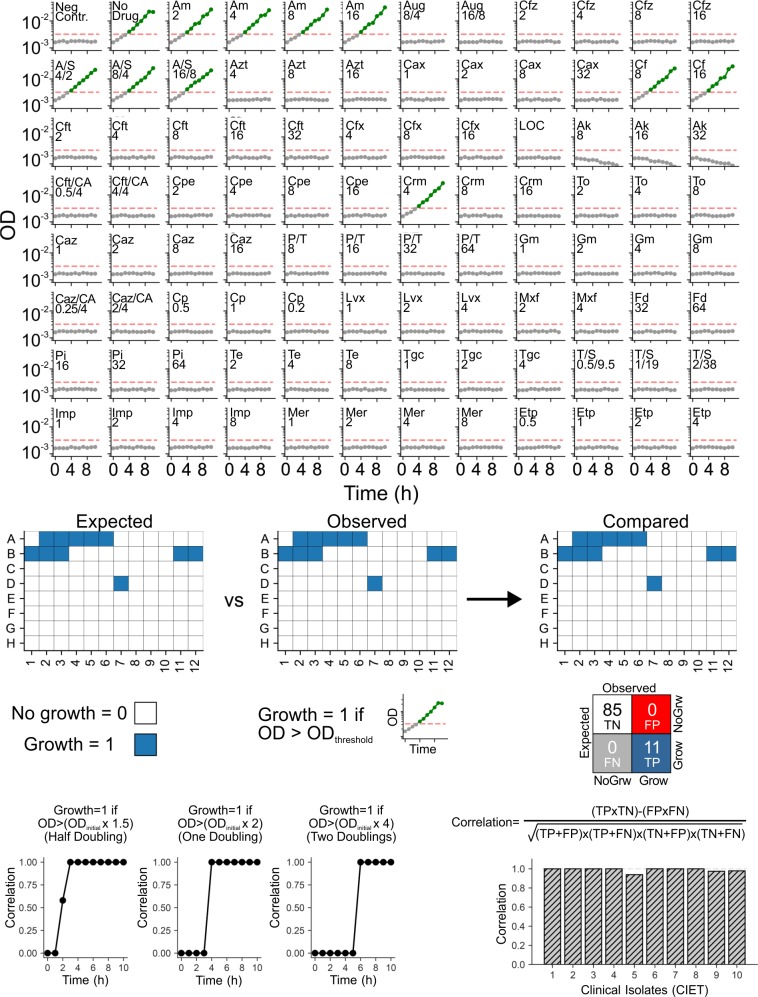
iFAST yields reliable results in shorter times in comparison to standard commercial clinical antibiotic susceptibility testing methods. (a) Utilizing the high sensitivity of the RUSD platform, iFAST can track growth of clinical isolates starting from the initial OD = 2 × 10^−3^ in antibiotic susceptibility test panels (NM43, Beckman Coulter). Cell densities of a clinical *E*. *coli* isolate (CIET-001) growing in different doses of 29 antibiotic compounds were periodically recorded at hourly intervals. Cell densities exceeding the growth threshold (4 × 10^−3^, horizontal red dashed lines, one doubling) are shown in green filled circles. Cell densities lower than the threshold are shown in gray filled circles. (b) Growth information was converted to a binary resistance map for the test panel, in which blue and white indicate growth and no growth in a given well, respectively. The Matthews correlation coefficient is calculated using the true–false matrix between observed and expected resistance data. (c) Correlation coefficient between iFAST measurements and clinical data changes as a function of growth threshold and hovers to acceptable levels as incubation times increase. For consistency and reliability purposes, we chose a 4 h incubation time and threshold of one population size doubling for the rest of the clinical *E*. *coli* strains. (Right panel) Histogram showing the correlations between iFAST measurements and clinical reports demonstrate the accuracy and power of the iFAST assay. (The data for Fig 4 can be found in S3 Data). Ak, amikacin; Am, ampicillin; A/S, ampicillin/sulbactam; Aug, amoxicillin/clavulanic acid; Azt, aztreonam; CA, clavulanic acid; Cax, ceftriaxone; Caz, ceftazidime; Cf, cephalothin; Cft, cefotaxime; Cfz, cefazolin; Cp, ciprofloxacin; Cpe, cefepime; Crm, cefuroxime; Etp, ertapenem; Fd, nitrofurantoin; FN, false negative; FP, false positive; Gm, gentamicin; iFAST, in Fiber Antibiotics Susceptibility Testing; Imp, imipenem; LOQ, limit of quantification; Lvx, levofloxacin; Mer, meropenem; Mxf, moxifloxacin; Neg Contr., negative control; OD, optical density; Pi, piperacillin; P/T, piperacillin/tazobactam; RUSD, rapid ultrasensitivity detector; Te, tetracycline; Tgc, tigecycline; TN, true negative; To, tobramycin; TP, true positive; T/S, trimethoprim/sulfamethoxazole.

Finally, we devised an antibiotic susceptibility assay to compare the RUSD method with the clinically approved standard antibiotic susceptibility tests, which typically start from higher cell densities (i.e., OD > 10^−3^). We utilized a commercially available gram-negative MIC panel (NM43, Beckmann Coulter B1017-420) that includes 29 antibiotics and antibiotic combinations (Table 1). We used 10 clinical *E*. *coli* isolates (CIET-001 to 010) that were previously phenotyped at the Clinical Microbiology Laboratory of Children’s Medical Center (CMLCMC), Dallas, Texas. For these measurements, we adhered to the manufacturer’s protocol for preparation and inoculation of the MicroScan Neg MIC Panel Type 43 (NM43, Beckman Coulter) in which a starting cell density of 0.5 McFarland (about 0.25 OD_600_) is then further diluted before inoculating into the antibiotic panel with a final target inoculum of 3–7 × 10^5^ CFU/mL. First, we grew every clinical strain for 24 h and determined MIC values by visual inspection following the guidelines provided by the manufacturer and confirmed that the MIC values we obtained were in line with the MIC values provided by the CMLCMC. Then, we grew one of the clinical strains (CIET-001) that was resistant to some of the compounds in the antibiotic panel, using different incubation times for optimizing minimum inhibition time required for MIC determination with RUSD (Fig 4A, Table 1). We performed 10 consecutive cell density measurements using RUSD at hourly intervals and tracked the growth in each well of the NM43 MIC test panel (Fig 4A). Using 3 different growth thresholds (half, one, and two doublings compared to initial inoculum size of about OD 2 × 10^−3^), we converted growth curves to binary resistance maps, in which blue indicates growth/resistance and white indicates inhibition/susceptibility (Fig 4B). This data set was compared to the expected resistance map for the same *E*. *coli* isolate. Correlations between the observed resistance map at different time points and the expected resistance map were calculated using the Matthews correlation coefficient $C=\frac{\left(TP\;x\;TN)-(FP\;x\;FN\right)}{\sqrt{\left(TP+FP)\;x\;(TP+FN)\;X\;(TN+FN)\;x\;(TN+FP\right)}}$ (Equation 3, in which TP is true positive, FP is false positive, TN is true negative, and FN is false negative). We also evaluated how this correlation changed as a function of growth threshold (Fig 4C). We found that for this particular strain (CIET-001), when a growth threshold of half doubling was considered, waiting 3 h or longer was enough to precisely reproduce the MIC values we obtained by 24 h incubation. Therefore, for consistency and improved accuracy, we set the growth threshold to one doubling (Fig 4A, dashed horizontal red lines, OD > 4 × 10^−3^) and incubation time to 4 h. Clinical isolates (CIET-002 to CIET-010) were tested by taking single measurements after 4 h of incubation. The correlation coefficient is 1.00 (perfect correlation) for 7 strains tested, with the remaining 3 isolates exceeding 0.9 (Fig 4C, right panel). Growth map for all 10 strains in (S5 Fig) shows the robustness with only 4 false positive (out of 950 measurements) reads. The complete MIC information for the 10 clinical isolates obtained with iFAST is provided in Table 1. Entries underlined and marked bold indicate disagreements with the clinical data. For example, we found the MIC for ampicillin for the CIET-10 strain as >16 μg/mL, whereas the clinical report suggested that it was ≤16 μg/mL. The breakpoint (BP) value for ampicillin for *E*. *coli* is 8 μg/mL. Since the AST panel we used cannot determine the exact MIC for ampicillin at this point, the discrepancy for this case might be considered as minor, as both tests indicate some resistance against ampicillin. In 5 other cases in which there are discrepancies between the NM-43 AST panel and iFAST, the errors for the cases of cefotaxime (BP = 0.25 μg/mL, iFAST MIC > 32 μg/mL, and NM-43 MIC ≤ 16 μg/mL), cefoxitin (BP 4 μg/mL, iFAST MIC ≤ 4 μg/mL, and NM-43 MIC > 16 μg/mL), ceftazidime (BP 0.25 μg/mL, iFAST MIC > 16 μg/mL, and NM-43 MIC > 8 μg/mL [CIET-5], ≤ 1 μg/mL [CIET-9]) could be classified as major errors. In the last case of ertapenem, we found an MIC ≤ 1 versus ≤ 2 by the plate method; however, the EUCAST system does not indicate a BP value for this drug. These differences might be due to experimental error or better sensitivity of our system. Finally, none of our measurements yielded false negative (FN) measurements when we missed growth of a bacterial strain in a drug condition in which it was expected to grow. These findings further articulate the power of the RUSD platform and the iFAST assay.

**Table 1:** MIC values obtained by iFAST and a standard clinical assay.

Drug (MIC values are in μg/mL)	Abbreviation	Breakpoint*	CIET-1	CIET-2	CIET-3	CIET-4	CIET-5	CIET-6	CIET-7	CIET-8	CIET-9	CIET-10
Source			Urinary tract infection	Urinary tract infection	Urinary tract infection	Urinary tract infection	Urinary tract infection	Blood infection	Blood infection	Urinary tract infection	Urinary tract infection	Urinary tract infection
Ampicillin	Am	***8***	>16	>16	>16	>16	>16	>16	>8	>16	>16	**>16 (≤16)**
Amoxicillin/clavulanic acid	Aug	***ND***	≤8/4	≤16/8	≤8/4	≤16/8	≤8/4	>16/8	>16/8	≤16/8	≤16/8	>16/8
Cefazolin	Cfz	***ND***	≤2	≤4	≤2	≤16	>16	>16	>16	>16	>16	>16
Ampicillin/sulbactam	A/S	***8***	>16/8	>16/8	≤4/2	>16/8	≤8/4	>16/8	>16/8	≤8/4	>16/8	>16/8
Aztreonam	Azt	***0*.*25***	≤4	≤4	≤4	≤4	>16	>16	>16	>16	≤4	>16
Ceftriaxone	Cax	***0*.*125***	≤1	≤1	≤1	≤1	>32	>32	>32	>32	≤1	>32
Cephalothin	Cf	***32***	>16	>16	≤8	≤8	≤8	≤8	≤8	≤8	≤8	≤8
Cefotaxime	Cft	***0*.*25***	≤2	≤2	≤2	≤2	**>32 (≤16)**	≤2	≤2	≤2	>32	>32
Cefoxitin	Cfx	***8***	≤4	≤4	≤4	≤4	≤4	≤4	≤4	≤4	≤4	≤4(>16)
Amikacin	Ak	***8***	≤8	≤8	≤8	≤8	≤8	≤8	≤8	≤8	≤8	≤8
Cefotaxime/clavulanic acid	Cft/CA	***0*.*25***	≤0.5/4	≤0.5/4	≤0.5/4	≤0.5/4	≤0.5/4	≤0.5/4	≤0.5/4	≤0.5/4	≤0.5/4	≤0.5/4
Cefepime	Cpe	***0*.*25***	≤2	≤2	≤2	≤2	>16	>16	>16	>16	≤2	>16
Cefuroxime	Crm	***8***	≤8	≤4	≤4	≤4	>16	>16	>16	>16	≤4	>16
Tobramycin	To	***2***	≤2	≤2	≤2	≤2	>8	≤2	≤2	>8	≤2	≤4
Ceftazidime	Caz	***0*.*5***	≤1	≤1	≤1	≤1	**>16 (>8)**	≤8	≤8	≤8	**>16 (≤1)**	≤1
Piperacillin/tazobactam	P/T	***8***	≤8	≤8	≤8	≤32	≤8	≤16	≤8	≤8	≤32	>64
Gentamicin	Gm	***2***	≤1	≤1	≤1	>8	>8	≤1	≤1	>4	≤1	>4
Ceftazidime/clavulanic acid	Caz/CA	***0*.*5***	≤0.25/4	≤0.25/4	≤0.25/4	≤0.25/4	≤0.25/4	≤0.25/4	≤0.25/4	≤0.25/4	≤0.25/4	>2/4
Ciprofloxacin	Cp	***0*.*064***	≤0.5	>2	>2	≤0.5	>2	>2	≤1	>2	≤0.5	>2
Levofloxacin	Lvx	***0*.*25***	≤1	>4	>4	≤1	>4	≤1	≤1	≤4	≤1	≤4
Moxifloxacin	Mxf	***0*.*25***	≤2	>4	>4	≤2	>4	≤2	≤2	>4	≤2	≤2
Nitrofurantoin	Fd	***64***	≤32	≤32	≤32	≤32	≤32	≤32	≤32	≤32	≤32	≤32
Piperacillin	Pi	***8***	≤16	>64	>64	>64	>64	>64	>64	>64	>64	>64
Tetracycline	Te	***8***	≤2	>8	≤2	>8	>8	≤2	≤2	≤2	≤2	≤8
Tigecycline	Tgc	***0*.*5***	≤1	≤1	≤1	≤1	≤1	≤1	≤1	≤1	≤1	≤1
Trimethoprim/sulfamethoxazole	T/S	***1***	≤0.5/9.5	>2/38	≤0.5/9.5	>2/38	≤0.5/9.5	>2/38	>2/38	≤0.5/9.5	>2/38	>2/38
Imipenem	Imp	***0*.*5***	≤1	≤1	≤1	≤1	≤1	≤1	≤1	≤1	≤1	≤1
Meropenem	Mer	***0*.*125***	≤1	≤1	≤1	≤1	≤1	≤1	≤1	≤1	≤1	≤1
Ertapenem	Etp	***ND***	≤0.5	≤0.5	≤0.5	≤0.5	≤0.5	≤0.5	≤0.5	≤0.5	≤0.5	≤1(≤2)

For a set of 29 antibiotics and/or antibiotic combinations. Bold and underlined text indicates the 4 cases in which iFAST indicated higher MIC concentrations than expected from the data (given in parenthesis).

*Breakpoint values are obtained from EUCAST MIC database.

Abbreviations: iFAST, in Fiber Antibiotics Susceptibility Testing; MIC, minimum inhibitory concentration; ND, no data.

## Discussion

The RUSD platform is a rapid, label-free, highly sensitive, and low-cost optical detection system that could be used as a field-deployable device. RUSD can detect about as few as 20–25 bacterial cells and a single fungal cell. RUSD can detect bacterial growth in less than an hour and quantitatively measure antibiotic susceptibility of pathogenic bacteria within 2 to 4 h using both small and large inoculum sizes. However, the use of RUSD will be particularly advantageous in cases for which the number of bacterial cells available are limited. RUSD can also be utilized to monitor contaminations in water or other fluids. In contrast to present technologies [10–12], RUSD can be easily assembled with readily available materials at a cost of about US$25 per device and a simple voltmeter device. In comparison to imaging-based or spectroscopic techniques, RUSD has higher throughput with higher sensitivity, without the need for image or signal processing. Here, we demonstrate RUSD as a viable platform that may be implemented into the current clinical framework of AST standards. Furthermore, the RUSD platform is amenable to technological modifications such as integration of advanced microfluidic and particle separation systems and portable electronics. The simplicity and the low-cost nature of RUSD will make it a viable diagnostic platform that can be employed in laboratories, hospitals, and industry.

## Materials and methods

### RUSD

RUSD is built around a hollow fused-silica optical fiber (500 μm diameter, 50–400 mm length, P.N. 25739 Supelco, n_i_ = 1.458). The hollow fiber is capped with a polydimethylsiloxane (PMDS)-glass assembly at both ends, each of which has a cavity cast using a small glass bead, connecting the hollow fiber and fluid inlet/outlet channels at 90° (Fig 1A). One side of the cavity in the PDMS layer is covered with a 1 mm thick soda lime glass, which creates a direct optical access into the fiber. The optical setup components include a 650 nm laser diode assembly with a focusing lens (5 mW, P. N. 0710893–000, AixiZ, Amazon.com) and a silicon photodiode (Digi-key 1080-1148-ND) as the detector. The current output from the detector is converted to voltage readings, which are recorded using a data acquisition card (DAQ card; MCC, 1408 HS) or a simple voltmeter and processed by a custom Matlab code. Code used for data acquisition is available on request. For the measurements with DAQ card, voltage is typically measured at 1,000 Hz sampling. Voltage is recorded for 10 s, and the median of these readings is used for OD measurements.

### Bacterial and fungal strains

Ten strains of *E*. *coli* (CIET-001 to CIET-010) isolated from pediatric patients (Children’s Medical Center, Dallas, TX, United States), 2 laboratory strains of *E*. *coli* (MG1655 and BW25113), a pan-sensitive clinical *E*. *coli* strain (ET-CI28) isolated from a pediatric patient, a laboratory strain of *P*. *aeruginosa* (PAO1, courtesy of Greenberg Lab, UTSW), and a laboratory strain of *S*. *aureus* (RN4220, courtesy of Marrafini Lab, Rockefeller University) were used in this study. Fungal strains used in this study were *C*. *glabrata* (ATCC 18126) and *S*. *cerevisiae* (S288c, courtesy of Springer Lab, Harvard Medical School).

### Calculating LOQ and LOD

For all cell density detection devices (including RUSD), we calculated LOD and LOQ values. Briefly, by measuring the corresponding OD or voltage readings for serially diluted cell cultures with known ODs and of multiple known blank media samples that contain no particles and/or cells, we generated calibration curves and identified LOD, which is the first data point with cells, where the measurement is significantly higher than the blank reading for LB media with no cells and can be reliably distinguished from the blank and at which detection is achievable. LOD was typically about 0.007 for our plate reader, about 0.002 for our spectrophotometer, and 5 × 10^−7^ for RUSD. These values improved slightly when a more transparent media was used. LOQ is the lowest concentration at which the cells can not only be reliably detected but at which our detection devices start responding proportionately with the sample density. LOQ was typically about 0.02 for our plate reader, about 0.006 for our spectrophotometer, and 1.5 × 10^−6^ for RUSD.

### Data analysis workflow

Voltage readings for serially diluted cell cultures with known ODs for the strain and media at hand were measured. Then, the recorded sample voltage was subtracted from the blank measurement voltage to obtain ΔV with respect to a blank measurement medium. This procedure was repeated for actual sample measurements. A calibration curve was plotted, and a ΔV to OD_600_ conversion function was generated using log–log regression. The LOD and LOQ were also calculated in this step using the log–log regression model and the recorded blank measurements. After running the samples through the device and collecting the voltage measurements, we converted the readings to corresponding OD_600_ using the conversion function. Then, any measurements below the LOQ were discarded to ensure that the analysis is based solely on statistically significant data.

### Cell counting with RUSD

Bacterial and fungal cells were diluted down to ODs nearly 10^−6^, which approximates the LOD for RUSD. RUSD is first blanked with growth media (LB for bacteria; yeast extract–peptone–dextrose [YPD] or yeast synthetic complete media for fungal cells). We then manually flow-diluted cell cultures and recorded corresponding OD values using RUSD. OD values were then converted to cell numbers using the following OD-to-CFU conversion factors: OD_600_ of 1 corresponded to approximately 5 × 10^8^ CFU/mL for *E*. *coli*, approximately 2.2 × 10^7^ CFU/mL for *S*. *cerevisiae*, and approximately 1.25 × 10^7^ CFU/mL for *C*. *glabrata*. Finally, calculated cell numbers were rounded to the closest integer. At very low cell densities approaching LOD, 5%–10% of the readings gave negative values such as −1 or −2, which we attributed to measurement noise or variability in blank measurements. These values were assumed to be zero in cell count histograms. Negative cell count values were not observed when cell densities were increased.

### Bacterial lag-time measurements

Isogenic bacterial cells were grown overnight in LB (RPI L24040-500.0) at 37°C with continuous shaking (220 rpm). OD (OD_600_) values for the overnight cultures were measured using a spectrophotometer (Eppendorf BioSpectrometer). Overnight bacterial cultures were serially diluted down to a cell density of 10^−6^ (OD_600_) in LB growth media in 96-well plates (12 replicates, 200 μL volume in each well), and cells were in an automated (temperature and humidity controlled) Tecan robotic system at 37°C with continuous shaking (about 200 rpm) for 24 h. OD of growing bacterial cultures was recorded approximately every 10 min. Lag times necessary for the cultures to reach OD_600_ of 0.02, the LOQ for our plate reader (Tecan Infinite M200), were calculated using a custom Matlab code. Code used for the analysis is available on request.

### AST for bacteria

Isogenic bacterial cells were grown overnight in LB at 37°C with continuous shaking (220 rpm), diluted to a final density of (OD_600_) 10^−6^ in 96-well plates, and grown in an increasing range of antibiotic concentrations. OD of these cultures was periodically (every 10–12 min) recorded by a plate reader (Tecan Infinite M200). Time-resolved dose-response curves were generated using OD readings. ODs that were significantly larger than 0.02 (Student *t* test, *p* < 0.05) were considered as growth for MIC calculations. Corresponding dose-response curves were analyzed for predicting MIC values using a custom Matlab code [13]. Antibiotics used for this study are levofloxacin (Alfa Aesar J66943), amikacin (Alfa Aesar J63862), and P-T (Apotex Corp #763).

### iFAST

Isogenic bacterial cells were grown overnight in LB at 37°C with continuous shaking (220 rpm) and were diluted to a final density of (OD600) 10^−6^ in glass flasks. These cultures were typically split into 6 subcultures (150 mL volume, LB media), and antibiotic solutions were added to each culture such that predicted MIC values were spanned across the flasks. Flasks were continuously shaken at 220 rpm at 37°C in a commercial shaker. Samples (approximately 1 mL were periodically taken from each culture every 30 min, and cell densities of bacterial cultures were recorded by RUSD and a spectrophotometer for comparison. Corresponding dose-response curves were analyzed for predicting MIC values using a custom Matlab code [13]. Code used for the analysis is available on request. LOQ and LOD were calculated for every experiment, and ODs that were significantly larger than LOQ (Student *t* test, *p* < 0.05) were considered as growth for MIC calculations. A calibration measurement was done before each iFAST test in order to calculate LOQ and LOD values.

### Clinical AST for bacteria using a commercial antibiotic panel

Clinical isolates were plated from −80°C stocks overnight on LB agar. Using a 1 μl inoculation loop, 5–6 isolated colonies were collected from the overnight plate. The bacterial mass was emulsified in 3 mL of sterile inoculum water (Beckmann Coulter B1015-2) by vortexing. Per the clinical standards, the final turbidity is adjusted to the equivalent of 0.5 McFarland standard, which corresponds to OD_600_ = 0.25 for *E*. *coli*. The standardized suspension (0.1 mL, or 100 μl) was transferred into 25 mL of inoculum water with PLURONIC (Beckmann Coulter B1015-7). The resulting inoculum was transferred into NM43 antibiotic susceptibility panel (by Beckmann Coulter B1017-420), with 0.2 mL in each well. The panels were covered with lids and incubated at 37°C without shaking in a humidified environment following instructions provided with the NM43 panels. The plates were visually checked 24 hr after inoculation per Beckman Coulter NM43 guidelines.

### Clinical AST for bacteria with NM43 MIC panel by iFAST

The bacterial response to the antibiotics on the NM43 panels were tracked using RUSD at hourly intervals. In order to track the susceptibility through a 10 h period, 10 panels were inoculated as described above and simultaneously started incubation. At the end of each interval, background and base signal levels were recorded. For the rest of the iFAST measurements, bacteria were grown in NM43 panels for 4 h, and cell densities were measured using RUSD. Cell densities exceeding 4 × 10^−3^ OD were considered to be positive for growth. In between each measurement, RUSD was flushed with DI water in order to avoid contamination and false positive measurements.

## Supporting information

S1 Fig Calibration measurements for RUSD. (a) Calibration measurements for *E*. *coli*, *S*. *aureus*, *P*. *aeruginosa*, and *S*. *cerevisiae*. (b) LOD (red dashed lines) and LOQ (blue dashed lines) of RUSD for a typical bacterial measurement. (b) Bacterial growth track of *E*. *coli* MG1655 at 37°C LB between OD = 1 × 10^−5^ and OD = 2 × 10^−3^. (The data for S1 Fig can be found in S4 Data). LB, lysogeny broth; LOD, limit of detection; LOQ, limit of quantification; OD, optical density; RUSD, rapid ultrasensitivity detector.
(TIF)

S2 Fig Lag time as a function of inoculum size. Lag time before any statistically significant growth as a function of inoculum size for *P*. *aeruginosa* (PA01), *S*. *aureus* (RN4220), *E*. *coli* (MG1655), and clinical isolate *E*. *coli* (ET-CI28). (The data for S2 Fig can be found in S5 Data)
(TIF)

S3 Fig Microbroth dilution assays to measure the first MIC values. Microbroth dilution assays performed using an automated robotic platform requires 7 to 14 h to yield the first MIC value. (a) Dose-response curves of levofloxacin for *P*. *aeruginosa*, *S*. *aureus*, and *E*. *coli*. (b) Comparison of dose-response curves of levofloxacin, amikacin, and piperacillin-tazobactam for *E*. *coli*. (The data for S3 Fig can be found in S6 Data). MIC, minimum inhibitory concentration.
(TIF)

S4 Fig MIC determination time comparisons of RUSD and plate reader. MIC determination time comparisons of RUSD and plate reader for (a) amikacin (ET-CI28) and (b) piperacillin-tazobactam (BW25113). (The data for S4 Fig can be found in S7 Data). MIC, minimum inhibitory concentration; RUSD, rapid ultrasensitivity detector.
(TIF)

S5 Fig Antibiotic susceptibility tests of clinical isolates using iFAST. Clinical isolates (CIET-001 to CIET-010) from −80°C stocks were plated on LB agar. Using a 1 μl inoculation loop, 5–6 colonies were collected and were emulsified in 3 mL of sterile inoculum water (Beckmann coulter B1015-2). The concentration was adjusted to OD = 0.2. The standardized suspension (100 μl) was transferred into 25 mL of inoculum water with PLURONIC (Beckmann Coulter B1015-7). The resulting inoculum was transferred into NM43 Antibiotic Susceptibility MIC Panel by Beckmann coulter, with 0.2 mL in each well. The panels were incubated at 37°C without shaking in a humidified environment. iFAST measurements were done after a 4 hr incubation period. In between each measurement, RUSD was flushed with sterile water in order to eliminate contamination. For each isolate, a resistance map was created and was compared to the expected resistance map by calculating Matthews correlation coefficient as described in the main text. (The data for S5 Fig can also be found in S3 Data). iFAST, in Fiber Antibiotics Susceptibility Testing; LB, lysogeny broth; MIC, minimum inhibitory concentration; OD, optical density; RUSD, rapid ultrasensitivity detector.
(TIF)

S1 Movie Progress of microbroth dilution assays to measure the first MIC values. MIC, minimum inhibitory concentration.
(MOV)

S1 Data Data file for Fig 2: Low bacterial and fungal cell counts taken by RUSD. RUSD, rapid ultrasensitivity detector.
(XLSM)

S2 Data Data file for Fig 3: Heat map plots of bacterial growth as a function of time and antibiotic dosage. (XLSM)

S3 Data Data file for Fig 4 for growth track of clinical isolates using iFAST in antibiotic susceptibility test panels. iFAST, in Fiber Antibiotics Susceptibility Testing.
(XLSM)

S4 Data Data for S1 Fig. Calibration measurements for RUSD. RUSD, rapid ultrasensitivity detector. (XLSM)

S5 Data Data for S2 Fig. Lag time as a function of inoculum size. (XLSM)

S6 Data Data for S3 Fig. Microbroth dilution assays to measure the first MIC values. MIC, minimum inhibitory concentration.
(XLSM)

S7 Data Data for S4 Fig. Antibiotic susceptibility tests of clinical isolates using iFAST. iFAST, in Fiber Antibiotics Susceptibility Testing.
(XLSM)

## Abbreviations

A/S: ampicillin/sulbactam
AST: antibiotic susceptibility testing
Aug: amoxicillin/clavulanic acid
Azt: aztreonam
BP: breakpoint
CA: clavulanic acid
Cax: ceftriaxone
Cf: cephalothin
Cfz: cefazolin
CMLCMC: Clinical Microbiology Laboratory of Children’s Medical Center
CFU: colony forming unit
Cp: ciprofloxacin
Cpe: cefepime
Crm: cefuroxime
FN: false negative
Fd: nitrofurantoin
Gm: gentamicin
iFAST: in Fiber Antibiotics Susceptibility Testing
Imp: imipenem
LOD: limit of detection
LOQ: limit of quantification
LB: lysogeny broth
Mer: meropenem
MIC: minimum inhibitory concentration
Mxf: moxifloxacin
ND: no data
Neg Contr.: negative control
n_i_: index of refraction
OD: optical density
P-T: piperacillin-tazobactam
RUSD: rapid ultrasensitive detector
T/S: trimethoprim/sulfamethoxazole
Te: tetracycline
Tgc: tigecycline
To: tobramycin
