# Supplementary figures and images for: Rapid ultrasensitive detection platform for antimicrobial susceptibility testing
Source: PLoS Biol. 2019 May 30;17(5):e3000291. doi: 10.1371/journal.pbio.3000291 (PMC6559665; doi:10.1371/journal.pbio.3000291)

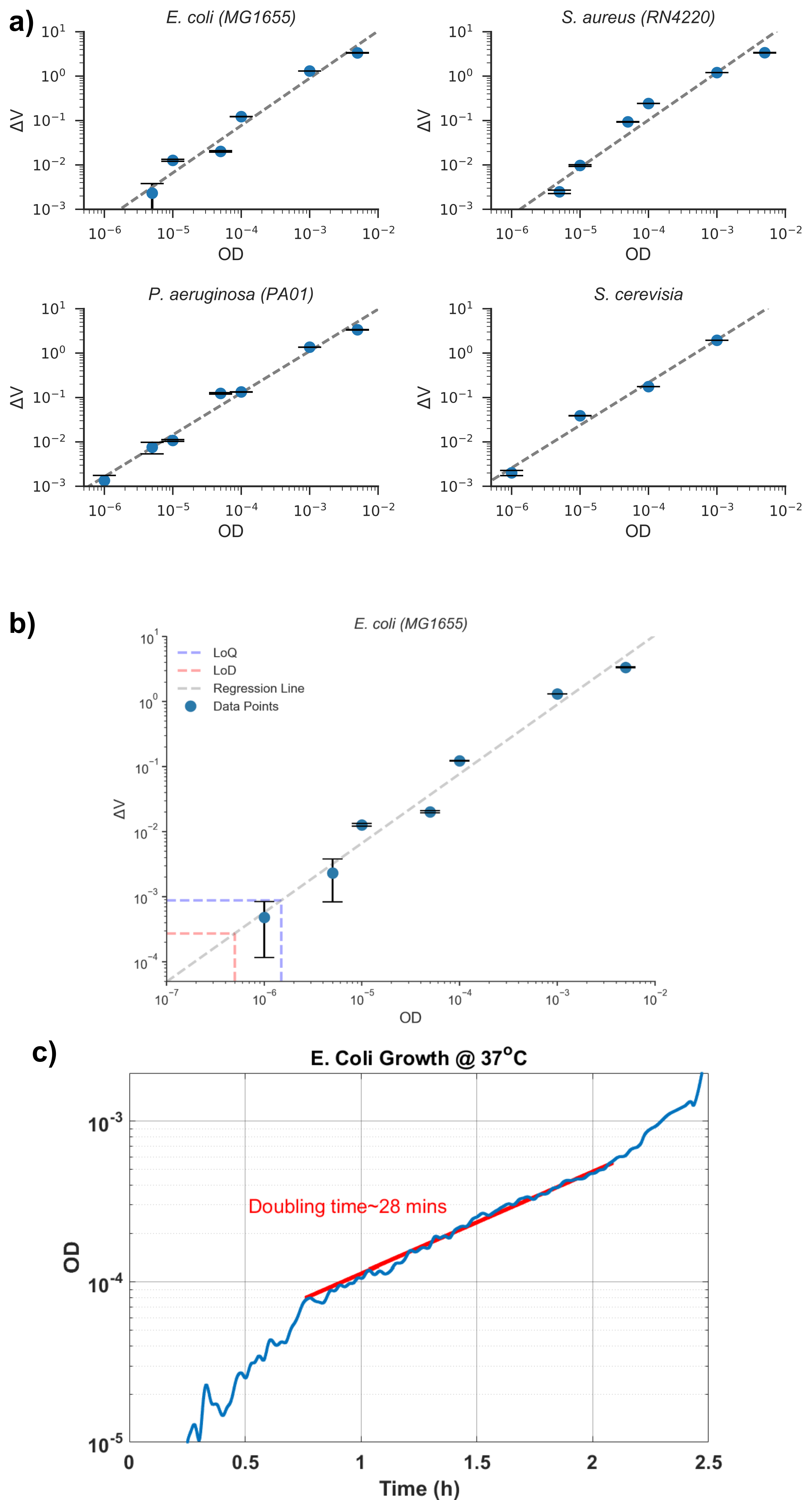

Supplement: S1 Fig — (a) Calibration measurements for E. coli, S. aureus, P. aeruginosa, and S. cerevisiae. (b) LOD (red dashed lines) and LOQ (blue dashed lines) of RUSD for a typical bacterial measurement. (b) Bacterial growth track of E. coli MG1655 at 37°C LB between OD = 1 × 10^−5^ and OD = 2 × 10^−3^. (The data for S1 Fig can be found in S4 Data). LB, lysogeny broth; LOD, limit of detection; LOQ, limit of quantification; OD, optical density; RUSD, rapid ultrasensitivity detector. (TIF) [file pbio.3000291.s001.tif]

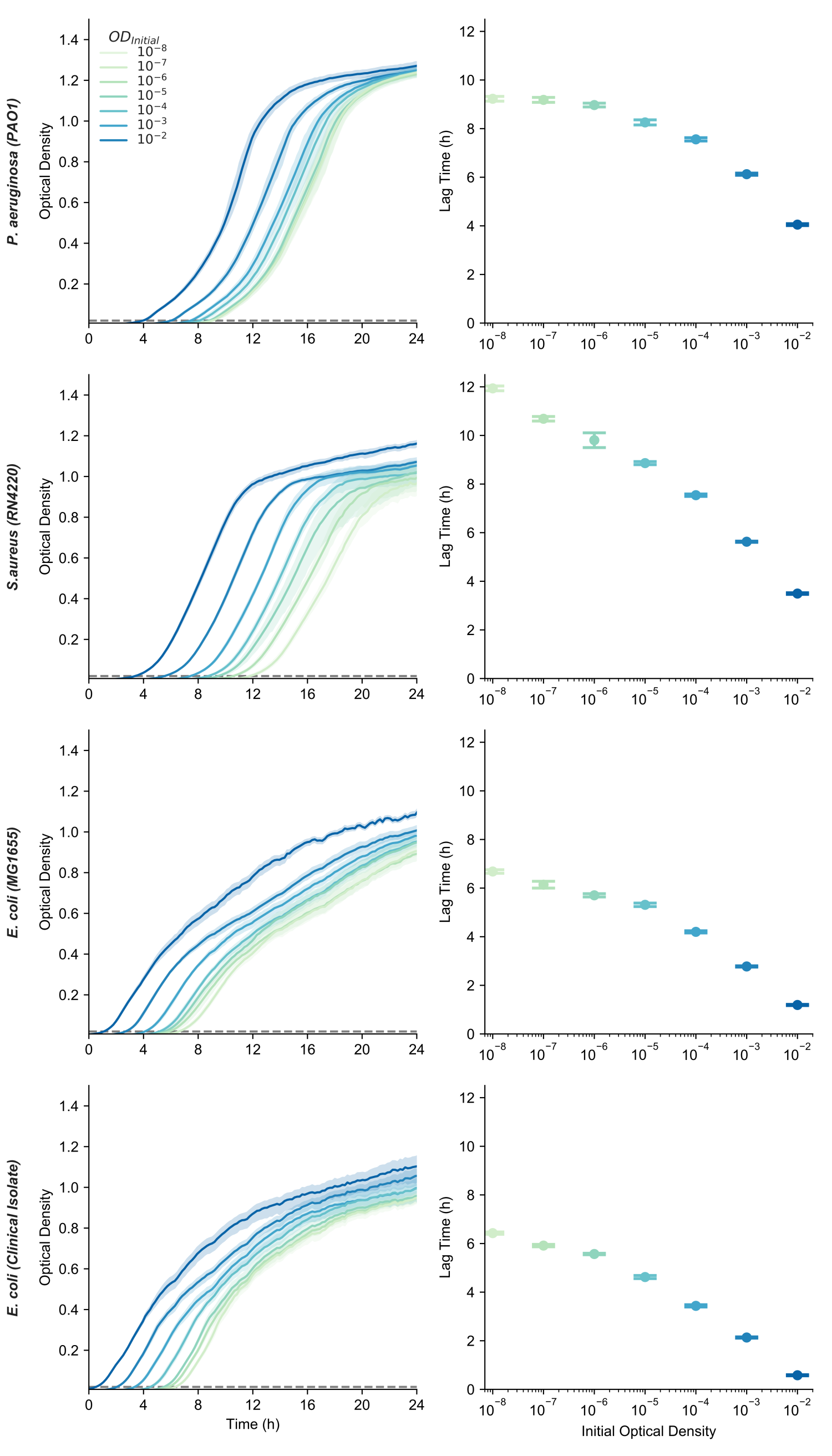

Supplement: S2 Fig — Lag time before any statistically significant growth as a function of inoculum size for P. aeruginosa (PA01), S. aureus (RN4220), E. coli (MG1655), and clinical isolate E. coli (ET-CI28). (The data for S2 Fig can be found in S5 Data) (TIF) [file pbio.3000291.s002.tif]

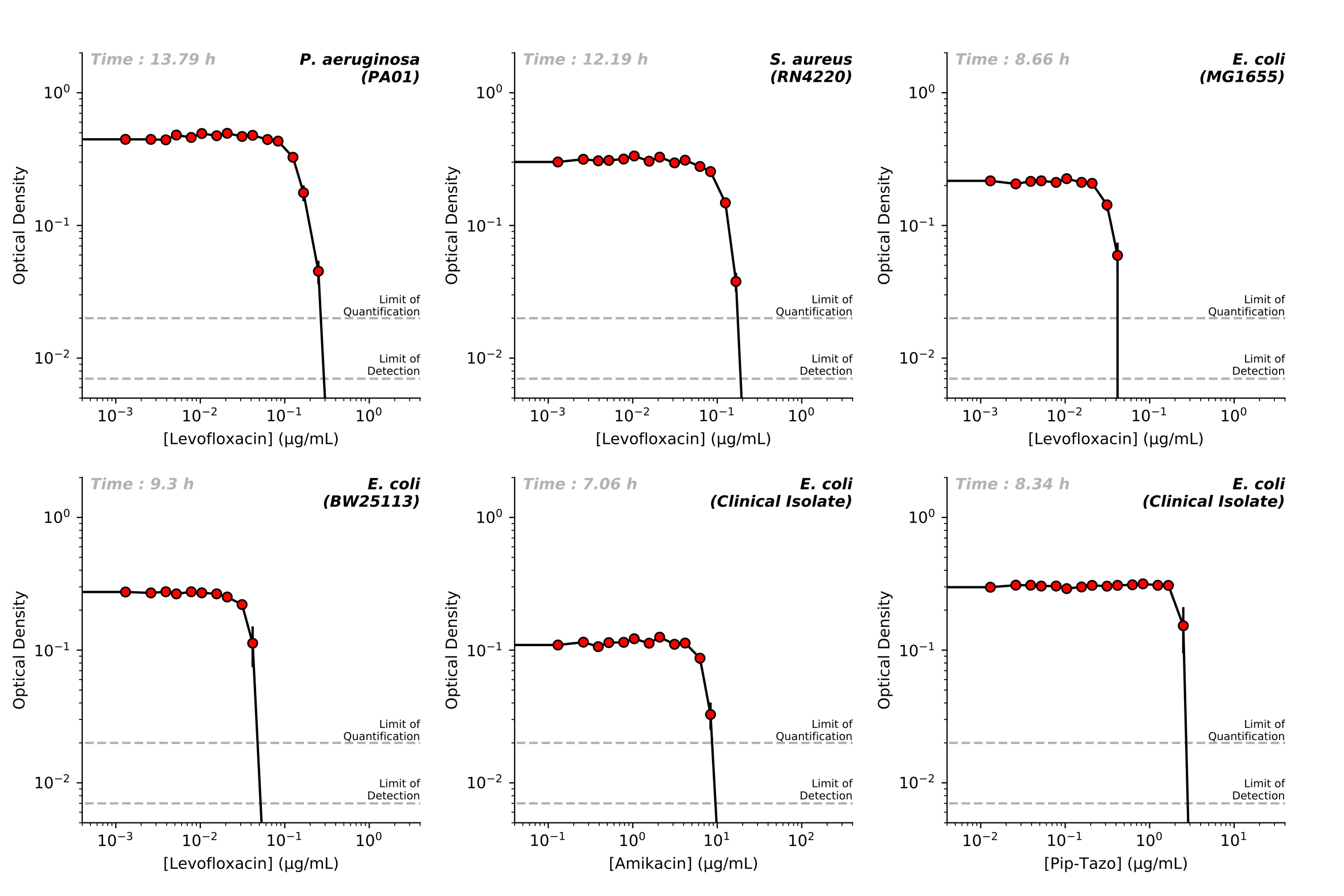

Supplement: S3 Fig — Microbroth dilution assays performed using an automated robotic platform requires 7 to 14 h to yield the first MIC value. (a) Dose-response curves of levofloxacin for P. aeruginosa, S. aureus, and E. coli. (b) Comparison of dose-response curves of levofloxacin, amikacin, and piperacillin-tazobactam for E. coli. (The data for S3 Fig can be found in S6 Data). MIC, minimum inhibitory concentration. (TIF) [file pbio.3000291.s003.tif]

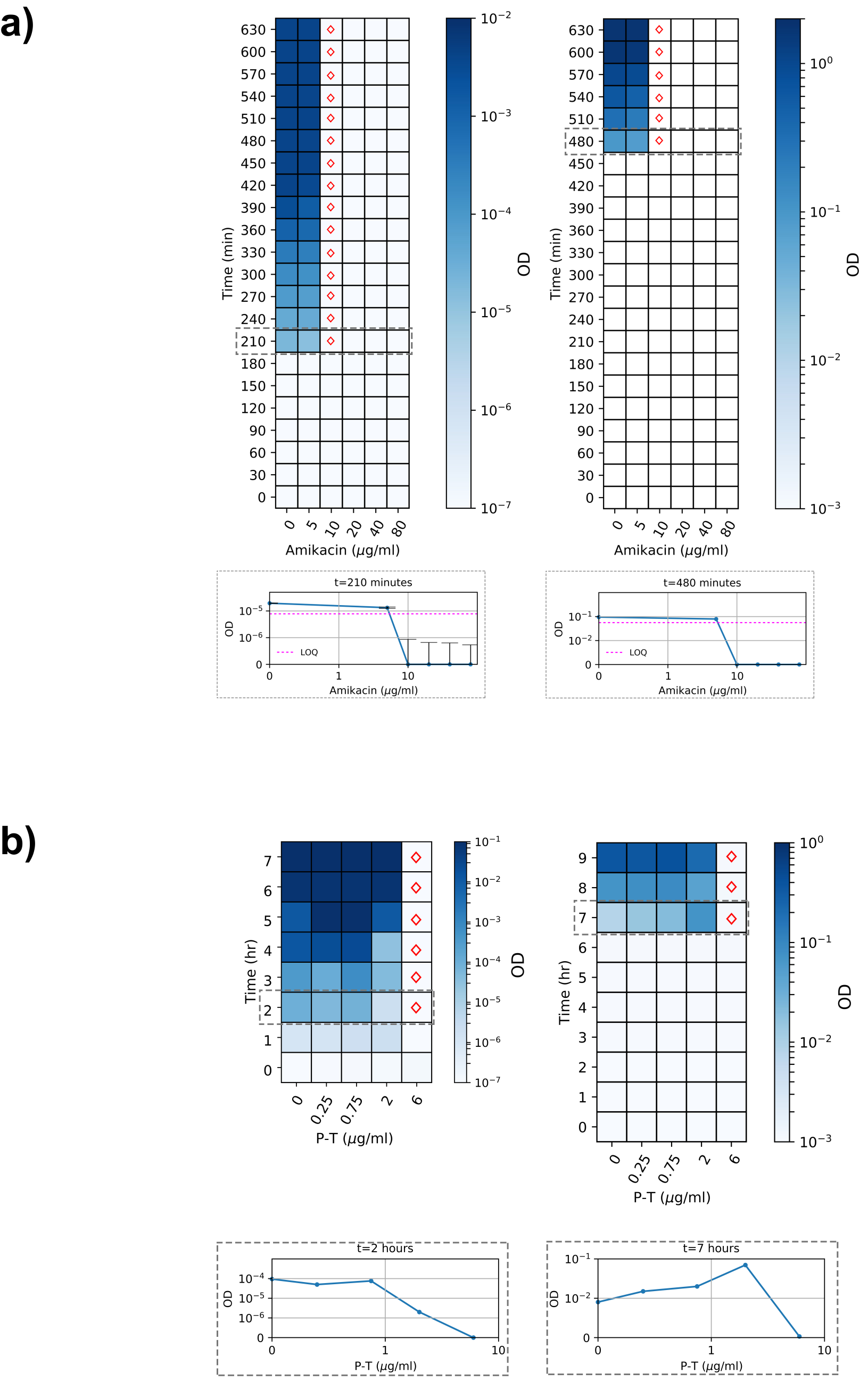

Supplement: S4 Fig — MIC determination time comparisons of RUSD and plate reader for (a) amikacin (ET-CI28) and (b) piperacillin-tazobactam (BW25113). (The data for S4 Fig can be found in S7 Data). MIC, minimum inhibitory concentration; RUSD, rapid ultrasensitivity detector. (TIF) [file pbio.3000291.s004.tif]

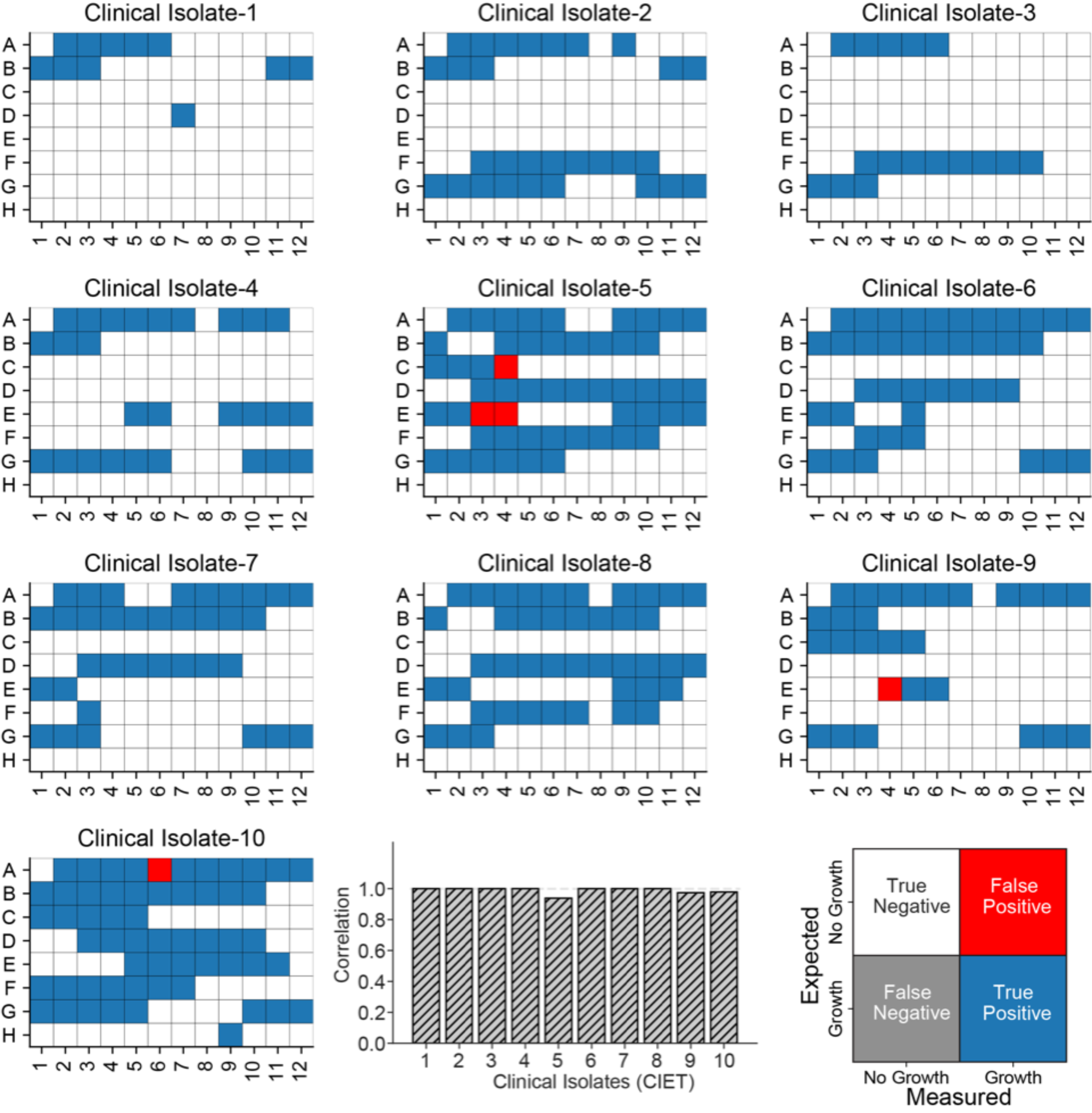

Supplement: S5 Fig — Clinical isolates (CIET-001 to CIET-010) from −80°C stocks were plated on LB agar. Using a 1 μl inoculation loop, 5–6 colonies were collected and were emulsified in 3 mL of sterile inoculum water (Beckmann coulter B1015-2). The concentration was adjusted to OD = 0.2. The standardized suspension (100 μl) was transferred into 25 mL of inoculum water with PLURONIC (Beckmann Coulter B1015-7). The resulting inoculum was transferred into NM43 Antibiotic Susceptibility MIC Panel by Beckmann coulter, with 0.2 mL in each well. The panels were incubated at 37°C without shaking in a humidified environment. iFAST measurements were done after a 4 hr incubation period. In between each measurement, RUSD was flushed with sterile water in order to eliminate contamination. For each isolate, a resistance map was created and was compared to the expected resistance map by calculating Matthews correlation coefficient as described in the main text. (The data for S5 Fig can also be found in S3 Data). iFAST, in Fiber Antibiotics Susceptibility Testing; LB, lysogeny broth; MIC, minimum inhibitory concentration; OD, optical density; RUSD, rapid ultrasensitivity detector. (TIF) [file pbio.3000291.s005.tif]
